# Amplidiff: an optimized amplicon sequencing approach to estimating lineage abundances in viral metagenomes

**DOI:** 10.1186/s12859-024-05735-4

**Published:** 2024-03-23

**Authors:** Jasper van Bemmelen, Davida S. Smyth, Jasmijn A. Baaijens

**Affiliations:** 1 Intelligent Systems Department, Delft University of Technology, Delft, Netherlands; 2 Department of Natural Sciences, Texas A &M University-San Antonio, San Antonio, TX USA; 3 Department of Biomedical Informatics, Harvard Medical School, Boston, MA USA

**Keywords:** Amplicon sequencing, Primer design, Abundance estimation, Set cover problem

## Abstract

**Background:**

Metagenomic profiling algorithms commonly rely on genomic differences between lineages, strains, or species to infer the relative abundances of sequences present in a sample. This observation plays an important role in the analysis of diverse microbial communities, where targeted sequencing of 16S and 18S rRNA, both well-known hypervariable genomic regions, have led to insights into microbial diversity and the discovery of novel organisms. However, the variable nature of discriminatory regions can also act as a double-edged sword, as the sought-after variability can make it difficult to design primers for their amplification through PCR. Moreover, the most variable regions are not necessarily the most informative regions for the purpose of differentiation; one should focus on regions that maximize the number of lineages that can be distinguished.

**Results:**

Here we present AmpliDiff, a computational tool that simultaneously finds highly discriminatory genomic regions in viral genomes of a single species, as well as primers allowing for the amplification of these regions. We show that regions and primers found by AmpliDiff can be used to accurately estimate relative abundances of SARS-CoV-2 lineages, for example in wastewater sequencing data. We obtain errors that are comparable with using whole genome information to estimate relative abundances. Furthermore, our results show that AmpliDiff is robust against incomplete input data and that primers designed by AmpliDiff also bind to genomes sampled months after the primers were selected.

**Conclusions:**

With AmpliDiff we provide an effective, cost-efficient alternative to whole genome sequencing for estimating lineage abundances in viral metagenomes.

## Background

Studying the composition of metagenomic samples through genome sequencing has led to insights into diverse microbial communities [1–3]. While whole (meta)genome sequencing is frequently used for this purpose, not all regions of the genome are equally informative. If the goal is to distinguish between different lineages, strains, variants, or species, we should focus on those regions that allow us to discriminate between the genomes of interest. For example, many studies focus only on 16S (bacteria and archaea) or 18S regions (eukaryotes) for metagenomic profiling [4]. Such a targeted sequencing approach has two main advantages: first, it is cost-efficient as it requires fewer sequencing reagents per sample, and second, it reduces the computational burden in downstream processing due to generating less data that needs to be processed.

Targeted sequencing, otherwise known as amplicon sequencing, is widely applied in the context of *Wastewater-Based Epidemiology* (WBE) [5–9]. In wastewater-based epidemiology, the objective is to study population-scale phenomena, such as trends in the number of infections based on corresponding pathogen concentrations found in wastewater samples. Since this method is non-invasive and captures information from the entire population within a geographical area, it is less prone to biases that occur in clinically obtained samples [10]. However, wastewater samples can suffer from low concentrations of genetic material, environmental RNA degradation, the presence of inhibitors, and high fragmentation rates [11]. For this reason, amplification of the genetic material of interest through Polymerase Chain Reaction (PCR) is a crucial step in wastewater analysis.

The importance of WBE itself has become apparent during the SARS-CoV-2 pandemic. A multitude of studies have confirmed that SARS-CoV-2 can be detected in wastewater samples and that we can estimate COVID-19 case numbers from such samples [12]. Moreover, we can estimate the relative abundances of different SARS-CoV-2 lineages (and therefore different variants) from wastewater samples using amplicon-based sequencing (an overview of relevant approaches is given in [13]).

The key step in performing amplicon-based sequencing is designing a set of primers such that the genetic material of interest can be amplified. Several physicochemical constraints (e.g. melting temperature, inter-primer interaction, intra-primer interaction) have to be considered, for which various primer design tools have been developed (e.g. Primer3 [14], Primer-BLAST [15], PriMux [16] and openPrimeR [17]). These tools take as input a set of reference genomes and a region of interest and attempt to find a set of primers such that the region of interest can be amplified in as many reference genomes as possible. In addition, PrimalScheme [18] and Olivar [19] have the option to find a near-minimal size set of primers such that the resulting amplicons would cover the entire genome, enabling amplicon-based Whole Genome Sequencing (WGS).

These primer design tools have been successfully applied in practice, but they can only find primers once a region of interest has been provided, or when the goal is to amplify the whole genome. This suffices when such regions are known a priori, for example in 16S sequencing [20] and 18S sequencing [21]. However, for viral genomes, this is not the case, and hence there is a need for tools that can find regions of interest, along with primers. When the goal is to estimate the relative abundances of strains or lineages present in a sample, primers should be designed such that the resulting amplicons can be used to differentiate. Since potential amplicons are only useable if we can find feasible primers in the flanking regions, primer and amplicon selection should be considered simultaneously. Yet, existing tools only consider one of these tasks, but not both.

Here we introduce a new methodology, AmpliDiff, that identifies discriminatory amplicons in a set of genomes originating from multiple lineages of a single species. Simultaneously, AmpliDiff finds primers in relatively conserved regions, such that the amplicons can be amplified through PCR. We demonstrate AmpliDiff by designing amplicons and corresponding primers for SARS-CoV-2 genomes. The effectiveness of these amplicons is assessed by evaluating the abundance estimation accuracy for different SARS-CoV-2 lineages on simulated data, comparing estimates on selected amplicons versus whole genome sequencing. Finally, by showing that these primers also match genomes discovered months after the primers were generated, we conclude that AmpliDiff provides a robust, efficient, and economic alternative to whole genome amplification in the context of viral metagenomic profiling.

## Results

### An overview of AmpliDiff

**Fig. 1:**
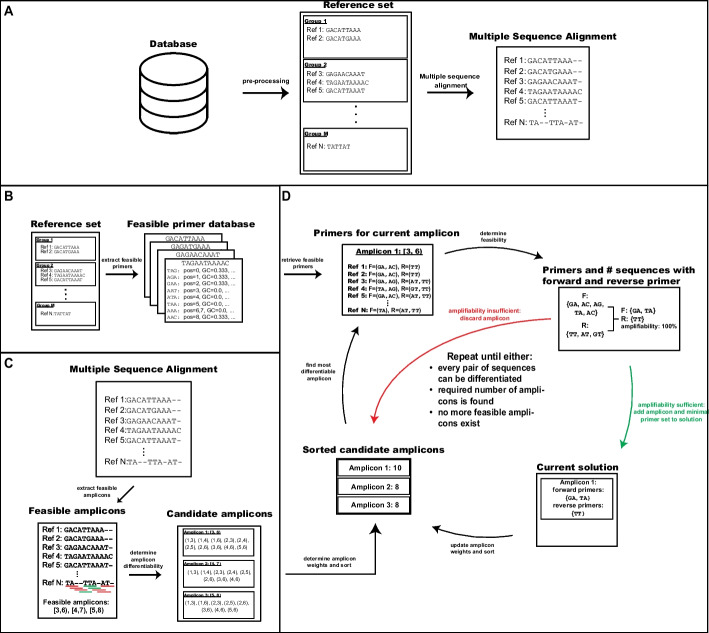
Outline of the workflow of AmpliDiff. **A** Pre-processing consists of building a reference set from a database of genomes (e.g. GISAID) and applying multiple sequence alignment to obtain multiple aligned genomes. **B** AmpliDiff builds a database of feasible primers that can be considered during the greedy amplicon selection (iii) step. **C** Feasible amplicon candidates are extracted from the multiple aligned genomes, and for every amplicon, the pairs of genomes it can differentiate are stored. **D** Greedy amplicon and primer selection until either all pairs of genomes can be differentiated, a user-defined number of amplicons is found, or no more feasible amplicons exist. AmpliDiff outputs the selected amplicons and corresponding primer sets

AmpliDiff is designed to find a minimal set of amplicons, along with corresponding primers, to differentiate between pre-defined groups in a given set of input genomes. The algorithm consists of three steps: (i) extracting a set of candidate primers, (ii) finding candidate amplicons and determining their ability to discriminate, (iii) greedily selecting amplicons and validating whether it is possible to find a set of primers for these amplicons. Figure 1 outlines the overall methodology of AmpliDiff which we will briefly describe here, with a more thorough explanation in the Methods section.

The input to AmpliDiff consists of multiple aligned genomes in the form of a *Multiple Sequence Alignment* (MSA), each with a corresponding group label. AmpliDiff then first extracts every physicochemically feasible primer of a given length and stores them in a *primer database* (Fig. 1B) to be used in step (iii). Next, candidate amplicons are found and their differentiability is determined (Fig. 1C). We define the *differentiability* of an amplicon as the number of pairs of genomes belonging to different groups that it can differentiate. In its final step, AmpliDiff heuristically tries to find a minimal set of amplicons with corresponding primers to differentiate between all the pairs of genomes. This is done by iteratively finding the most differentiable amplicon and checking if it is possible to find forward and reverse primers to amplify it in the input genomes (Fig. 1D), given the physicochemical constraints (see Methods).

Defining the *amplifiability* of an amplicon as the relative number of genomes in which both a forward and reverse primer flanking the amplicon can be found, AmpliDiff only accepts amplicons that meet a user-provided minimal required amplifiability (default = 95%). If this amplifiability is met, AmpliDiff minimizes the number of primer pairs needed to amplify the amplicon and adds the amplicon along with a minimal set of primers to the solution. Afterward, the differentiability of the remaining candidate amplicons is updated to correct for the pairs of genomes that can already be differentiated, and the amplicons are sorted (highest to lowest) based on the newly calculated differentiabilities. When the amplifiability of an amplicon is too small, the amplicon is discarded and the next most differentiable amplicon is considered. This cycle is repeated until either every pair of genomes can be differentiated, a predefined number of amplicons has been found, or there are no more candidate amplicons.

The final output of AmpliDiff consists of the resulting set of amplicons (with coordinates based on the provided MSA) along with the corresponding forward and reverse primers. Throughout this paper, we consider a minimal required amplifiability of 95%.

### AmpliDiff finds highly differentiable amplicons in SARS-CoV-2 genomes

Using AmpliDiff we have determined two sets (length 200 and 400 bp, respectively, corresponding to Illumina iSeq and MiSeq sequencing) of 10 amplicons on a collection of SARS-CoV-2 genomes. As input, we used a global reference set of 2749 SARS-CoV-2 genomes over all 1837 different lineages (1–7 genomes per lineage) that existed at the time that these sequences were downloaded (18 August 2022).

We define the groups for AmpliDiff to distinguish between as the SARS-CoV-2 lineage labels assigned to genome sequences. The reference set was constructed by following the pre-processing steps described in the VLQ pipeline [22], using all of the available genomes on GISAID [23], and were multiple aligned with MAFFT [24]. Every genome on GISAID has a Pango lineage [25] label. These lineages consist of closely related SARS-CoV-2 genomes which are defined by key phylogenetic markers and shared mutations. Our objective is to find amplicons that allow us to differentiate between these lineages.

**Fig. 2:**
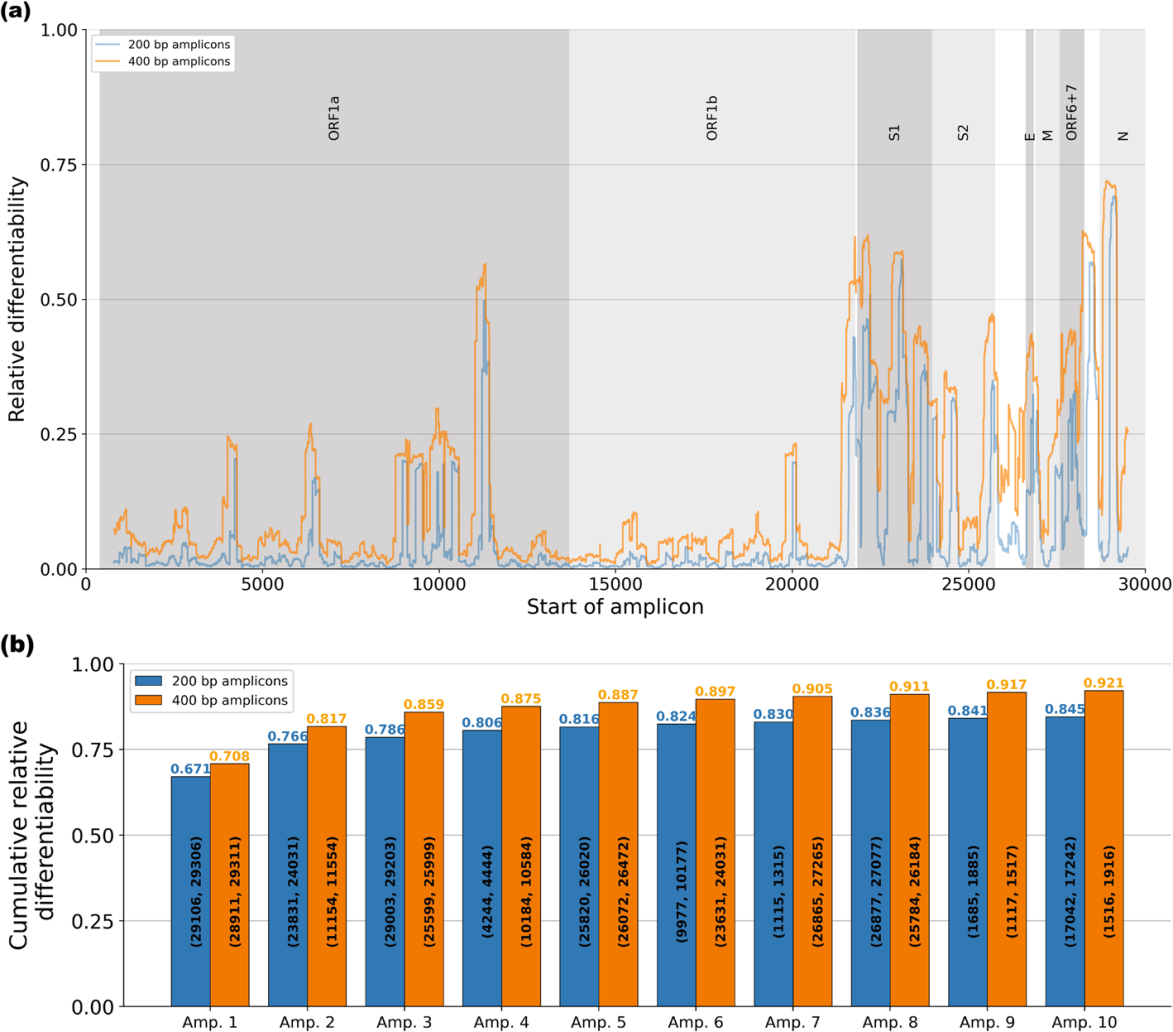
Overview of the differentiability of potential amplicons, and the amplicons selected by AmpliDiff on the global SARS-CoV-2 dataset. **a** Relative differentiability of all amplicons of widths 200 bp (blue) and 400 bp (orange) that have at least 50 nucleotides before or after them. The shaded areas correspond to the annotations in the top of the figure.**b** Cumulative relative differentiability of the ten best amplicons (200 and 400 bp, respectively) found by AmpliDiff

Figure 2a shows the relative differentiability, defined as the differentiability of an amplicon divided by the total number of pairs of genomes belonging to different groups, for all 200 bp and 400 bp amplicons with at least 50 bp flanking on either side. Note that any 200 bp amplicon contained by a 400 bp amplicon, is at most as differentiable as the 400 bp amplicon. We observe that both the ORF1a and ORF1b regions are relatively conserved between the reference genomes and that regions close to the 3’-end (right) of the genome are generally more differentiable than regions closer to the 5’-end (left), with individual amplicons reaching up to 71.96% differentiability. However, it is not guaranteed that every amplicon is practically feasible since we need primers flanking the amplicons in order to amplify these regions through PCR.

The amplicons selected by AmpliDiff and their cumulative relative differentiability are shown in Fig. 2b. For both amplicon widths, we see that the first amplicon is chosen in the gene that encodes the nucleocapsid protein (N), which provides a relative differentiability of 67.1% and 70.8% for amplicons of 200 and 400 bp, respectively. Subsequent amplicons are spread across the genome, but in both cases, we observe that there is a large overlap in genomic areas covered by selected amplicons. Moreover, Table S1 in the Additional file 1 shows that, for a minimal required amplifiability of 95%, most amplicons require only a single primer pair, whereas higher thresholds necessitate more primer pairs.

We also observe that for both the 200 and 400 bp amplicons, the added differentiability suffers from diminishing returns when adding amplicons. The greedy algorithm selects amplicons that are most differentiable, and the first 5 amplicons already allow us to differentiate between 81.6% and 88.7% of the pairs of genomes of different lineage, for widths 200 and 400 bp, respectively. Any further amplicons contribute approximately 0.4–1% to the cumulative relative differentiability indicating that, depending on the required resolution, it can be sufficient to use up to 5 amplicons. Similar results were obtained for different minimal required amplifiabilities as shown in Figures S1–S6 in the Additional file 1.

### Benchmarking AmpliDiff amplicons versus WGS

We show the effectiveness of amplicons selected by AmpliDiff by comparing abundance estimation results for SARS-CoV-2 lineages based on AmpliDiff amplicons, to abundance estimation results based on whole genome sequencing. To this end, we have generated two datasets: one for Texas and one for the Netherlands. Both datasets consist of a *reference set* (different from the reference set used for AmpliDiff) which contains the genomes that the abundance estimator uses as reference material, and a *simulation set* that contains genomes to simulate reads from. For both datasets, the reference genomes consist of genomes available on GISAID for the corresponding location, sampled between May and October of 2022. The simulation sets consist of genomes from the corresponding location with a sampling date in November 2022. A more detailed explanation of the setup for the simulation study can be found in the Methods section.

We perform abundance estimation using VLQ [22], a pipeline for estimating the relative abundance of viral lineages from wastewater sequencing data. VLQ employs Kallisto [26] to assign a relative abundance to every genome in a set of reference genomes, based on the likelihood that reads originate from that genome. The relative abundance of an entire lineage is then obtained by aggregating the relative abundances of all genomes that are part of the lineage. To evaluate the outcome of a simulation we calculate the corresponding *Mean Absolute Scaled Error*, which is defined as $\text {MASE}:= \frac{\frac{1}{|\mathcal {L}|} \sum _{l \in \mathcal {L}} |\phi _l - \hat{\phi }_l|}{\frac{1}{|\mathcal {L}|} \sum _{l \in \mathcal {L}} |\phi _l - \overline{\phi }|}$. Here, $\phi _l$ and $\hat{\phi }_l$ are the true abundance and estimated abundance of a lineage $l \in \mathcal {L}$ respectively ($\mathcal {L}$ being the set of all lineages), and $\overline{\phi }$ is the average true abundance of lineages.

For both locations, we simulated sequencing data for amplicon widths of 200 and 400 bp, using the top 1, 2, 5, and 10 amplicons, or the whole genome. For each setting, we repeated the read simulation 20 times with different random seeds. For the amplicons selected by AmpliDiff, we check whether the corresponding primers bind (i.e. match exactly) to the input genomes—any sequences that do not have matching primers are not amplified, so we do not simulate reads in this case (see Methods). It is challenging, however, to maintain a similar approach for the whole genome-based simulations. There, we assume that both read coverage and read depth are uniform over the simulation genomes, and we do not check for primer binding. From a practical point of view, this is an ideal but highly unrealistic situation, particularly if we consider the context of wastewater sequencing [22]. Hence, the whole genome-based results should be considered as idealized results only serving as a benchmark for the amplicon-based abundance estimations.

**Fig. 3:**
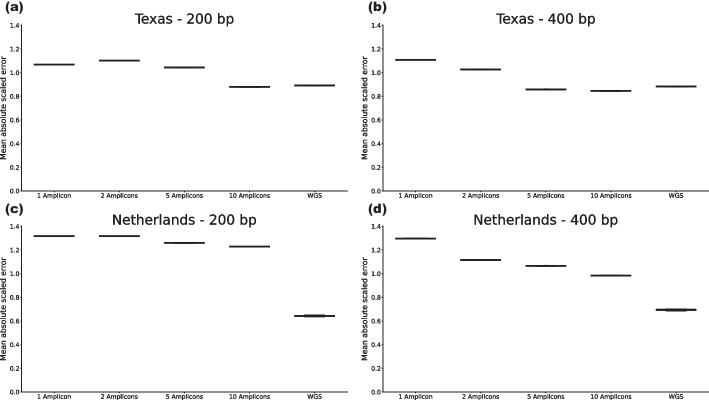
Distribution of mean absolute scaled errors of abundance estimations in both datasets for both amplicon widths using varying numbers of amplicons. **a** Texas dataset using amplicons of width 200. **b** Texas dataset using amplicons of width 400. **c** Netherlands dataset using amplicons of width 200. **d** Netherlands dataset using amplicons of width 400

Figure 3 shows the MASEs of abundance estimations for all experiments. In this figure we observe that the abundance estimations show very little variance, indicating that results are consistent in all configurations and that the MASEs range between 0.6 and 1.3 with the whole genome-based approach usually achieving the smallest MASE. Moreover, in the amplicon-based results we see that adding amplicons, in general, reduces the MASE, and that for the Texas dataset using 5 or 10 amplicons of width 400 bp, or 10 amplicons of width 200 bp, the amplicon-based approach outperforms the whole genome-based approach. Given that the whole genome simulated data reflects a strongly idealized setting, these results highlight the power of careful amplicon selection by AmpliDiff.

In contrast to the Texas dataset, we see a substantial discrepancy between amplicon-based and whole genome-based estimation results in the Netherlands dataset. This is particularly prominent for the amplicons of width 200 bp where, even with 10 amplicons, the whole genome-based approach strongly outperforms the amplicon-based approach. This can be explained by the limited discriminatory power of short amplicons, or because of primers not binding (which is not taken into account in the idealized WGS setting).

Alternatively, this discrepancy can be due to the fact that nearly all of the genomes used in the simulation study are (sub)lineages or recombinant lineages of the B.1.1.529 lineage (Omicron). Omicron sublineages can be particularly hard to distinguish in metagenomic samples as they are highly similar. Moreover, as most sublineages of B.1.1.529 in the simulation datasets did not exist in the PANGO nomenclature when the global reference set was constructed, the AmpliDiff amplicons were not explicitly designed to differentiate between these sublineages. When we correct for this by changing the resolution (i.e. by aggregating sublineages of B.1.1.529), we see that amplicon-based results substantially improve, generally becoming comparative or better than whole genome-based results (Figure S8 in the Additional file 1).

### AmpliDiff is robust against missing data

When designing reference sets informative reference genomes may be left out, either due to limitations in computational resources or because of scarcity in the available information. Here we consider what happens when AmpliDiff is presented with an incomplete reference set and the effect it has on the amplicons selected by AmpliDiff. For this, we take 15 random subsamples of sizes 1500 (54.6%), 2000 (72.8%), and 2500 (90.9%) respectively from the reference set used here (2749 reference genomes) and run AmpliDiff with the same settings as before.

**Fig. 4:**
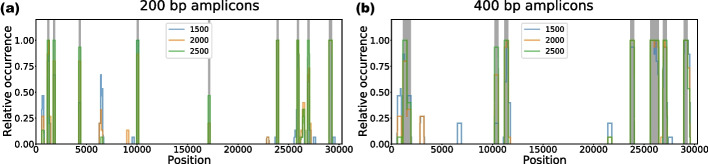
Assessment of the robustness of amplicons selected by AmpliDiff through randomly sampling subsets of reference genomes of sizes 1500, 2000, 2500, each repeated for 15 random seeds. **a** shows for every position (in the aligned genomes) how often it was contained in an amplicon of width 200 in each of the subsampling experiments, with grey regions indicating the amplicons on the full reference set, whereas **b** shows the same results for amplicons of width 400

Figure 4 shows how often a nucleotide position is covered by an amplicon in a subsample, relative to the total number of subsamples, for amplicons of 200 bp and 400 bp width. We observe that, regardless of the number of excluded reference sequences and amplicon length, the majority of the results agree on which areas of the genome to cover. Moreover, the regions that are covered in at least 80% of these experiments also appear to overlap with the initial amplicons selected based on the full reference set. Any variation in covered nucleotide positions stems from amplicons selected in the later stages of the greedy cycle. These amplicons generally contribute only marginally to the total differentiability of the selected amplicons. Moreover, in these later stages, there are often multiple candidate amplicons with similar differentiability. Nevertheless, these results indicate that AmpliDiff is robust against missing reference genomes, even when nearly half of the reference genomes are excluded.

### Primers and amplicons found by AmpliDiff remain viable over time

A crucial aspect of amplicon design is that the primers also bind to genomes that are not present in the dataset used to determine the amplicons. We can check how well the amplicons determined by AmpliDiff work on ‘new’ genomes, by checking for every amplicon whether both a forward and reverse primer of the corresponding primer set bind. As a source of new genomes, we have downloaded high-quality genomes from GISAID from September 2022 to May 2023 (while the AmpliDiff input genomes were sampled up to August 2022) and checked the amplifiability of every amplicon in these genomes.

**Fig. 5:**
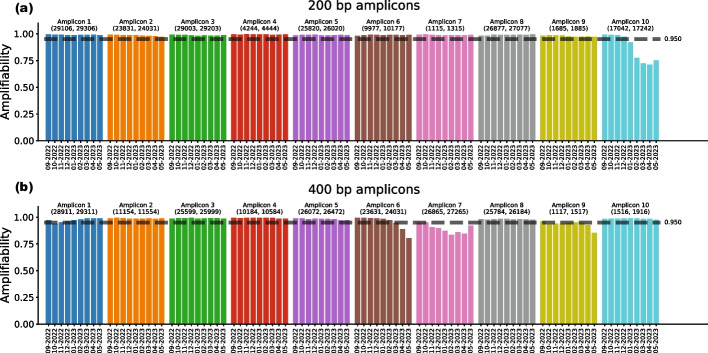
Fraction of genomes per month in which amplicons bind. **a** Amplifiability of 200 bp amplicons in the genomes from September 2022 to May 2023. **b** Amplifiability of 400 bp amplicons in the genomes from September 2022 to May 2023

Figure 5 shows the amplifiability per month of every selected amplicon (widths 200 and 400 bp). We observe that every 200 bp amplicon retains an amplifiability of at least 95% in every month, with the exception of the final amplicon (17,042–17,242). This amplicon, located in ORF1b, becomes less amplifiable over time, reaching an amplifiability of approximately 70% in April 2023. As this is the final amplicon selected by AmpliDiff, this will not have a significant effect on the ability to perform abundance estimation using the selected amplicons.

For amplicons of width 400 bp (Fig. 5b) we see more variation in amplifiability, particularly in amplicons 6, 7, and 9. As this again affects the later, less differentiable amplicons, we also expect that this does not strongly affect the abundance estimation accuracy using these amplicons. Results for different minimal required amplifiability are shown in Figures S9–S14 in the Additional file 1, where we see similar trends.

### Computational requirements

All results in this paper were obtained by running AmpliDiff on a High-Performance Computing cluster, using 200 GB of RAM and 12 CPUs. The total computation times for 200 bp and 400 bp amplicons using all 2749 sequences were 24,994 and 28,576 s wall clock time, respectively. This is mostly due to the greedy amplicon selection, which took 23,651 (94.6%) and 27,014 (94.5%) seconds, respectively. We observe similar behavior for fewer input sequences (Table S2 in the Additional file 1). The bottleneck for the greedy algorithm is finding exact solutions to the primer feasibility and minimization problems, which we expect to be NP-hard due to their relation to the set cover problem.

## Discussion

Here, we introduced AmpliDiff, a computational tool designed to find discriminatory genomic regions along with primers, with the objective of discriminating between different groups of genomes. AmpliDiff does not require prior knowledge of the input genomes beyond their respective lineages, and it avoids relying on pre-defined regions of interest such as the 16S rRNA gene in bacteria and archaea [27], or the internal transcribed spacer (ITS) region in fungi [28]. By focusing only on discriminatory genomic regions within a viral species, AmpliDiff provides an effective and economic alternative to amplification-based whole genome sequencing in the context of viral metagenomic profiling.

Through a simulation study, we have shown that the amplicons selected by AmpliDiff can be used to obtain accurate and consistent abundance estimates for SARS-CoV-2 genomes. Moreover, we found that abundance estimation using AmpliDiff amplicons can generally achieve similar accuracy as abundance estimation based on whole genome sequencing, despite only using up to 10 amplicons. This is a major improvement over whole genome-based amplification for SARS-CoV-2, which requires approximately 100 amplicons, and hence at least as many primer pairs.

Although AmpliDiff is based on a reference database, we see that amplicons selected by AmpliDiff remain highly discriminatory, even when the reference set used to determine the amplicons is not up to date. In particular, we observe that lineages get redefined over time, leading to misleading input for AmpliDiff: the amplicon selection approach may fail to distinguish between sequences from different lineages, as these were initially assigned to the same lineage. Hence, results could improve even further if the lineage assignment at the time of amplicon selection is stable.

A limitation of our benchmarking study is that we consider an idealized scenario for whole genome amplification, with complete genome coverage and uniform sequencing depth. While the simulated data for AmpliDiff amplicons takes the possibility of primers not binding (and hence amplicon dropout) into account, the simulated whole genome data does not. In our experiments, we check for exact primer binding, which the commonly used ARTIC primers are not designed for, and under these conditions, the ARTIC v4.1 amplicons would not amplify. If we were to incorporate less stringent primer binding requirements, we expect to see two effects: 1) AmpliDiff amplicons will amplify better, likely improving results, and 2) the WGS-based results would suffer from amplicon dropout, which results in decreased performance for the WGS-based approach.

The next step is to show experimentally that the AmpliDiff primers work in practice. This could be validated through in vitro experiments using synthetic populations and spiked-in samples of known variant composition. This would allow for a fair comparison against abundance estimation based on ARTIC primers, or other schemes, and to verify the effectiveness of primers and amplicons found by AmpliDiff in the context of SARS-CoV-2.

In addition, we aim to apply AmpliDiff to sequence databases for other viruses, such as Influenza viruses, SARS-CoV-1, poliovirus, and HIV. A prerequisite for any such analysis is a database with sufficiently many high-quality genomes. By using AmpliDiff for different species, we will gain additional insights into AmpliDiff’s effectiveness and limitations, allowing for further improvements to our methodology.

Finally, there are algorithmic aspects in AmpliDiff that can be improved. For example, the input genomes are pre-processed using MSA; since this is an NP-hard problem, we use a heuristic which can lead to sub-optimal alignments which in turn can affect amplicon selection. Moreover, MSA is not well suited for genomes with extensive recombination or repetitiveness, hence AmpliDiff is expected to work less well for complex genomes. Alternatively, pangenome representations (e.g. variation graphs [29] or colored de Bruijn graphs [30]) could serve as a data structure for AmpliDiff to work with. Moreover, our primer feasibility algorithm relies on solving a mixed integer linear program related to the set cover problem, which is therefore expected to be NP-hard. To this end, it would be valuable to prove hardness results for the amplicon selection problem and simultaneously work on finding dedicated algorithms for solving it efficiently.

## Conclusions

With AmpliDiff we introduce a computational tool to find genomic regions along with primers that can be used in the context of metagenomic profiling. Through a simulation study on SARS-CoV-2, we have shown that using amplicons found by AmpliDiff gives accurate and consistent abundance estimates of SARS-CoV-2 lineages, competitive with results based on WGS. Thus, AmpliDiff provides an effective and economic alternative to whole genome-based metagenomic profiling of diversity within a viral species.

## Methods

### AmpliDiff methodology

The input to AmpliDiff consists of a reference set of multiple aligned genomes $\mathcal {G}$, an amplicon width $L_A$, a primer width *k*, a primer search window length $L_0$, a maximum misalignment character threshold $L_{\emptyset }$, a maximal mismatch tolerance *D* and a minimal amplifiability threshold $\gamma$. In addition, AmpliDiff also accepts a list of groups for every genome in $\mathcal {G}$ in TSV format (otherwise AmpliDiff assumes that the objective is to discriminate between all pairs of input genomes), a trade-off parameter $\beta$ that is used to model the trade-off between adding primer pairs and the additional sequences in which an amplicon can be amplified, and finally a set of physicochemical properties that the primers have to satisfy. As shown in Fig. 1, AmpliDiff consists of three steps: (i) a pre-processing step that builds a database of feasible primers for the reference genomes, (ii) an amplicon pre-processing step that extracts feasible amplicons and calculates their differentiability, and (iii) a greedy amplicon selection procedure which iteratively finds the most differentiable amplicon and adds it to the solution if primers can be found to amplify the amplicon. Here we describe these steps.

#### Step (i): constructing the feasible primer database

The objective of AmpliDiff is to find amplicons along with primers such that they can be amplified with PCR. For this, it is necessary to consider the potential primer candidates that can be used from the genomes in $\mathcal {G}$. AmpliDiff considers all *k*-mers from the reference genomes as primer candidates, where *k* is the desired primer length. The primer candidates are filtered according to nucleotide content, melting temperature, risk of hairpin formation, and risk of self-dimerization (exact criteria are listed in the Additional file 1), which can be directly estimated from the *k*-mers. These properties impact the effectiveness of primers [31] and are therefore also considered in state-of-the-art primer design tools, such as Primer3 [14], OpenPrimeR [17] and PriMux [16]. To prevent unwanted byproducts during PCR, primers are also required to match the reference genomes in a unique location. Any *k*-mers that do not meet these criteria are marked as infeasible by AmpliDiff and removed from the primer database.

Unlike conventional primer design tools, AmpliDiff allows reference genomes to contain degenerate nucleotides and disambiguates them by considering every possible representation of the *k*-mer. If a degenerate *k*-mer has more than 1024 representations (user-defined threshold, by default 1024), it is immediately removed from the database. Step (i) results in a set of non-degenerate primers to be considered in subsequent steps of AmpliDiff.

#### Step (ii): amplicon pre-processing

By requiring that the input genomes $\mathcal {G}$ are multiple aligned, we ensure that similar regions in the genomes are aligned. Moreover, the resulting sequences in $\mathcal {G}$ have the same length, denoted $L_G$. We will adhere to the following notation. Given a genome $g \in \mathcal {G}$, we define *g*[*i*] as the nucleotide at position *i* in genome *g* (starting with index 0). Similarly, for non-negative integers $i < j \le L_G$, we let $g[i:j] = g[i,i+1,\ldots ,j-1]$ denote the substring of *g* starting at position *i* and ending before position *j*. Using this notation and the fact that all genomes in $\mathcal {G}$ have the same length, we can define an amplicon *A* as an interval $[i:i+L_A]$, where $L_A$ is the length of the amplicon. To further simplify notation, we will write $g[A]:= g[i:i+L_A]$ to refer to the substring defined by the interval $A = [i:i+L_A]$, in genome *g*.

In order for multiple aligned genomes to have the same length, they are padded by gap characters (‘-’). A gap character indicates that a genome has a gap at a given position, relative to (some of) the genomes it is aligned against. Multiple sequence alignment is an NP-hard problem under general conditions [32] and hence, most multiple sequence alignment programs (e.g. MAFFT [24] or ClustalW [33]) use heuristics. In practice, these heuristics generally provide sub-optimal alignments that have additional gaps, resulting in discriminatory regions based on falsely identified differences.

With AmpliDiff we combat this problem in two ways. The first is by using a threshold $L_{\emptyset }$ (default value is 10% of the amplicon length) for the maximum number of gap characters allowed in an amplicon, in all of the reference genomes. In addition to preventing amplicons that have an inflated number of gap characters due to sub-optimal multiple alignment, this also ensures that expected PCR products will have approximately the same length. The second way of handling the consequences of sub-optimal alignments is by incorporating a maximal mismatch tolerance *D* (default value is 0) that controls when AmpliDiff considers a pair of genomes to be differentiated by an amplicon. Specifically, two genomes *g* and $g'$ are considered differentiated by an amplicon *A* if and only if the Hamming distance between *g*[*A*] and $g'[A]$ is larger than *D*. In the presence of degeneracy, we only consider positions where there is no overlap between the possible base representations in the two genomes.

We apply another constraint on gap characters to ensure that there is a suitable region for primer selection flanking potential amplicons. Specifically, we require that every amplicon is preceded and directly followed by at least $L_0$ non-gap characters in every input genome. Together with the gap threshold condition, this yields a set of candidate amplicons which we will denote $\mathcal {A}$. For each candidate amplicon, we calculate its differentiability, i.e. the number of pairs of genomes belonging to different groups that can be differentiated by the amplicon, and store this for use in subsequent steps.

#### Step (iii): greedy amplicon selection

In the final step, AmpliDiff finds a near-minimal set of amplicons with corresponding primers, to differentiate between as many pairs of genomes belonging to different groups. The methodology employed by AmpliDiff is based on casting the amplicon finding problem as the *Set Cover Problem* (SCP) when looking to differentiate between all pairs of genomes from different groups, or the *Maximum Coverage Problem* (MCP) when looking to find a fixed number of amplicons that differentiate between as many pairs of group disjoint genomes as possible. These coverage problems are well-studied NP-hard combinatorial optimization problems [34, 35]. Instances of these coverage problems are given by a set of elements $\mathcal {U}$, called the universe, a collection of subsets of the universe $\mathcal {C} \subseteq 2^{\mathcal {U}}$ such that $\cup _{S \in \mathcal {C}}S = \mathcal {U}$, and a positive rational cost function $c\ :\ \mathcal {C} \rightarrow \mathbb {Q}_{++}$ that assigns a cost to every set in $\mathcal {C}$. In the SCP the objective is to find a minimal cost sub-collection $C^{*} \subseteq \mathcal {C}$ such that $\cup _{S \in \mathcal {C}^*} = \mathcal {U}$, whereas in the MCP the objective is to find a sub-collection of at most a given size covering as many elements of $\mathcal {U}$ as possible.

To see how these coverage problems relate to the amplicon selection problem, we can define our universe $\mathcal {U}$ as the set of all pairs of genomes belonging to different groups. Then, defining an amplicon as the subset of pairs of genomes it can differentiate between and assigning every amplicon a cost of 1, we directly obtain an instance of the SCP, or an instance of the MCP (Maximum Coverage Problem) if a maximum number of allowed amplicons is also given. As a consequence, we can use algorithms that have specifically been developed for these types of problems [36–40], to solve our amplicon finding problem.

For practical use, we also need to consider whether an amplicon can be amplified in sufficiently many genomes. Specifically, we require that an amplicon $A \in \mathcal {A}$ has both a corresponding forward and reverse primer in at least $\gamma \%$ of the input genomes. Since we do not know a priori which amplicons are able to satisfy this condition, this has to be determined for every amplicon. It is computationally infeasible to do this beforehand for all candidate amplicons because we also need to check primer interactions (see below). Therefore, we resort to a greedy approach similar to the greedy algorithm for the SCP and MCP [37]. Our approach iteratively adds the amplicon with the largest number of uncovered elements to the solution and whenever an amplicon is considered, we determine its amplifiability to see if it exceeds the minimal amplifiability condition. Note that the greedy algorithm for the SCP and MCP has been shown to be an approximation algorithm with approximation factors $\left( 1 + ln(|\mathcal {U}|)\right)$ and $(1 -\frac{1}{e})$ respectively, which means that we obtain an approximately optimal solution for our problem as well.

The greedy amplicon selection step in AmpliDiff works as shown in Fig. 1D. First, the candidate amplicons are sorted based on their differentiability (highest to lowest), and the most differentiable amplicon is picked, with ties broken arbitrarily. Using the primer database built in step (i), AmpliDiff finds feasible primers flanking this amplicon in all of the input genomes by solving a *Mixed Integer Linear Program* (MILP). The MILP is used to determine if it is possible to find primers that guarantee that the amplicon can be amplified in at least $\gamma \%$ of the input genomes (solving is done through Gurobi [41]). The MILP also checks whether it is possible to find enough forward and reverse primers that have approximately the same melting temperature (maximum difference of $5^\circ$ Celsius by default) and do not pose a significant risk of having primer-primer interactions. Full details can be found in the Additional file 1.

When an amplicon does not meet the minimal amplifiability requirement, it is discarded and the next most differentiable amplicon is considered. When an amplicon does meet the requirements, we solve the same MILP again, but now with the objective of finding a minimal set of primer pairs that enable the amplification of this amplicon in at least $\gamma \%$ of the genomes. Since the objective is to differentiate between pairs of genomes from different groups, we should prioritize finding primers for genomes that contribute most to the differentiation power of the amplicon. Generic primer design tools do not consider this and thus we cannot use these to find primers.

To solve this, we have adapted the objective function of the primer minimization problem: we simultaneously minimize the number of primer pairs required to amplify a candidate amplicon and maximize the number of pairs of amplified genomes differentiated by the amplicon (see Additional file 1). The trade-off between these conflicting criteria is modeled through a parameter $\beta$ (default value 5%), which allows adding primer pairs only if they increase the realized differentiability (i.e. differentiability of pairs of genomes in which the amplicon is amplifiable) by at least $\beta \%$ of the total differentiability of the amplicon. When the minimal required amplifiability is set to 100%, this trade-off is omitted since the amplicon is already required to be amplifiable in every input genome, and the number of required primer pairs is directly minimized instead.

After solving the primer minimization problem, the amplicon, along with the computed minimal set of primer pairs, is added to the solution and the remaining amplifiability of amplicons is updated. Any amplicons with zero differentiability are removed and candidate amplicons are again sorted based on their differentiability. The amplicon selection cycle continues until either all pairs of genomes belonging to different groups can be differentiated, a given maximum number of amplicons have been found, or no more feasible amplicons exist. AmpliDiff outputs the selection of amplicons and corresponding primer pairs.

### Assessment on SARS-CoV-2

#### Amplicon generation

We have generated sets of 10 amplicons of different widths and with different minimal amplifiability requirements by using a global reference set of SARS-CoV-2 genomes. This reference set was constructed from all available genomes on the GISAID database [23] up until 18 August 2022, by following the reference set construction procedure detailed in [22]. This resulted in a set of 2,749 reference genomes distributed over 1,837 Pango lineages with 1–7 genomes per lineage. The reference genomes were multiple aligned using MAFFT [24] to obtain the input genomes for AmpliDiff. We ran AmpliDiff with $L_A \in \{200, 400\}$, $k=25$, $L_0=50$, $L_{\emptyset }=0.1 \cdot L_A$, $D=1$, $\gamma \in \{90\%, 92.5\%, 95\%, 97.5\%, 99.9\%, 100\%\}$, $\beta \in \{5\%, 10\%\}$ and a maximum primer degeneracy of 1024. We obtained twelve sets of 10 amplicons of widths 200 and 400 bp and minimal amplifiabilities of 90%, 92.5%, 95%, 97.5%, 99.9%, and 100%, respectively ($\beta$ did not affect results). The resulting amplicons can be found in Figures S1–S6 in the Additional file 1.

#### Simulation study

To evaluate how well amplicons and primers selected by AmpliDiff work in the context of lineage abundance estimation, we have created two benchmarking datasets: one with genomes from the Netherlands (constructed on 13 April 2023) and another with genomes from Texas (constructed on 6 March 2023). We estimate relative abundances of different lineages in these datasets using the amplicons selected by AmpliDiff (95% minimal required amplifiability), as well as for whole genome sequencing, by applying the VLQ pipeline [22]. Both datasets consist of a *reference dataset* with genomes spanning the months of May until October 2022 that is used by VLQ as references for the abundance estimations, and a *simulation dataset* with genomes from November 2022. The simulation dataset is used to simulate reads for each region, and every simulation is repeated 20 times with different random seeds amounting to a total of 40 amplicon-based simulations and 40 whole genome-based simulations for each region.

All datasets are filtered based on completeness and coverage to include only genomes with at least 29,000 nucleotides which have at most 1% N-content and have fewer than 0.05% unique amino acid mutations (corresponding to the “complete” and “high coverage” filters on GISAID). The reference genomes were filtered further by following the VLQ pre-processing steps (see above), with the adjustment that they were not checked for ambiguous nucleotides.

For the whole genome-based abundance estimations, the reference genomes are directly used in VLQ. For the amplicon-based abundance estimation, we further process these reference genomes by extracting amplicons that are considered amplifiable in the respective genomes (see next subsection). We combine amplicon sequences per reference genome by concatenating them with sequences of 200 ‘A’ nucleotides in between. This ensures that only amplicon reference sequences are considered for abundance estimation while avoiding falsely mapping reads across amplicons.

Read simulations were done with ART [42], using the default mode for the whole genome-based reads and the amplicon mode for the amplicon-based reads. We have only simulated reads from amplicons that were amplifiable given the selected primers, whereas reads for the whole genome data were simulated uniformly over the simulation genomes. For the 200 bp amplicons, we simulated $2\times 150$ bp Illumina HiSeqX TrueSeq sequencing reads with an average fragment length of 200 bp. Similarly, for the 400 bp amplicons, we simulated $2\times 250$ bp Illumina MiSeq v3 reads with an average fragment length of 400 bp. For the whole genome-based reads we applied the same settings, with the standard deviation of the fragment size set to 10 bp. For a fair comparison, we simulate datasets with a similar number of reads, hence we set the read depth to 1000$\times$ for the amplicon-based read simulations, and to 100$\times$ for the whole genome-based simulations. The lineage abundances in the simulated samples reflect the relative abundances of lineages in the simulation datasets.

#### Primer binding and amplicon extraction

In our experiments, we assume that primers only bind to genomes if they match a genome exactly, as this is how primers are generated during step (i) of AmpliDiff. In practice, it is possible that primers bind to genomes in the presence of nucleotide mismatches, so this provides a conservative estimate of how amplifiable the amplicons found by AmpliDiff are. We do allow up to 10 degenerate nucleotides in a primer binding site, where a primer is considered to bind if one of the respective representations matches the corresponding nucleotides in the primer. We thereby avoid discarding potential primer-binding sites due to the presence of degenerate nucleotides.

### Supplementary information

**Additional file 1.** Supplementary Figures S1–S14, Supplementary Tables S1–S2, Primer selection criteria, and Primer feasibility/optimization model.

## Data Availability

The reference genomes used for determining the AmpliDiff amplicons are available through GISAID under identifier EPI_SET_230825gp. The reference genomes and genomes used to simulate reads from the Texas-based simulation experiments are available through GISAID under identifiers EPI_SET_230825zm and EPI_SET_230825pe, respectively. The reference genomes and genomes used to simulate reads from the Netherlands-based simulation experiments are available through GISAID under identifiers EPI_SET_230825he and EPI_SET_230825fe, respectively. The simulated sequencing data (.fastq files) for the 95% amplifiability amplicons and whole genome sequencing approaches are available on Zenodo (https://zenodo.org/record/8298887) [43]. The code for AmpliDiff can be found on Github (https://github.com/JaspervB-tud/AmpliDiff) under the MIT license [44]. AmpliDiff relies on Gurobi [41], a commercial solver which is free for academic use.
