# Supplementary material for: Amplidiff: an optimized amplicon sequencing approach to estimating lineage abundances in viral metagenomes
Source: BMC Bioinformatics. 2024 Mar 23;25:126. doi: 10.1186/s12859-024-05735-4 (PMC10960382; doi:10.1186/s12859-024-05735-4)
Supplement: Supplementary file 1 — Additional file 1. Supplementary Figures S1–S14, Supplementary Tables S1–S2, Primer selection criteria, and Primer feasibility/optimization model. [file 12859_2024_5735_MOESM1_ESM.pdf]

Supplementary Information

Cumulative differentiability and primer selection

The amplicons considered in the main text are based on a minimal required amplifiability of 95%. Here we present the results for amplicons picked for minimal required amplifiabilities of 90%, 92.5%, 95%, 97.5%, 99.9% and 100%. The resulting cumulative differentiabilities for every respective configuration can be found in Figures S1-S6. For minimal required amplifiabilities below 99.9% we see that roughly the same regions were selected, wheras for 99.9% and 100% the strictness in requirements become a bottleneck and different amplicons are chosen. Nevertheless, we see that the initial amplicon is always located in the nucleocapsid gene and that the first few amplicons contribute the most to the cumulative differentiability.

Interestingly, it can be seen that in both the 99.9% and 100% minimal required amplifiability results, the first amplicon has a larger relative differentiability than the first amplicons for lower minimal required amplifiabilities. This is due to the fact that our primer minimization model includes a trade-off between the number of primer pairs selected and the additional differentiability that is gained by adding them. Therefore, even though any solution allowed for higher minimal required amplifiability requirements is also feasible for lower requirements, the model can decide to not add primer pairs (and therefor make the amplicon not amplifiable in all genomes), if they allow for only a marginal increase in differentiability. Hence, the same amplicon can achieve slightly lower differentiability.

The impact of requiring a higher minimal amplifiability on the number of selected primer pairs can be seen in Table S1. In the table we show the minimal number of forward and reverse primers required to amplify an amplicon found by AmpliDiff. Results are mostly similar for minimal required amplifiabilities below 97.5%, where generally a single primer pair is needed for the amplification of an amplicon in the input genomes. For minimal required amplifiabilities of at least 97.5%, however, we see that the number of required forward and reverse primers increases substantially, requiring up to 13 forward primers or 9 reverse primers at a minimal required amplifiability of 100%.

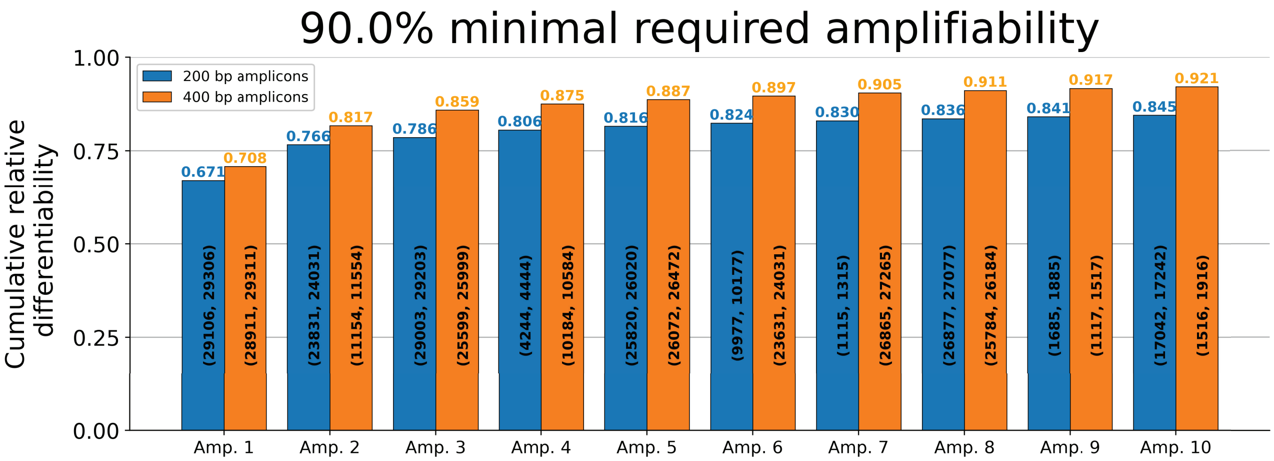

Figure S1: Cumulative relative differentiability of ten best amplicons (both 200 and 400 widths) found by AmpliDiff with the requirement of at least 90% amplifiability

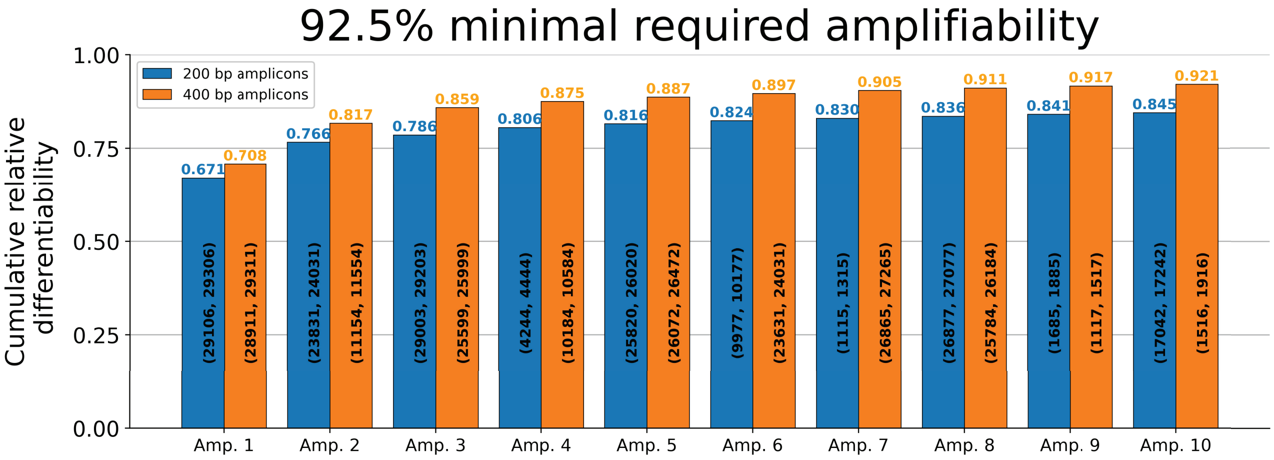

Figure S2: Cumulative relative differentiability of ten best amplicons (both 200 and 400 widths) found by AmpliDiff with the requirement of at least 92.5% amplifiability

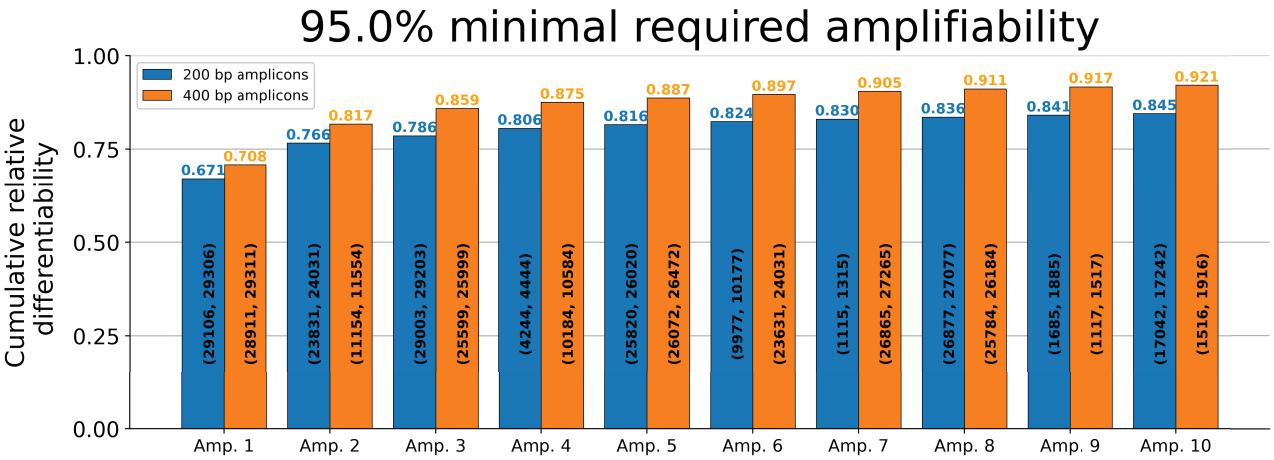

Figure S3: Cumulative relative differentiability of ten best amplicons (both 200 and 400 widths) found by AmpliDiff with the requirement of at least 95% amplifiability

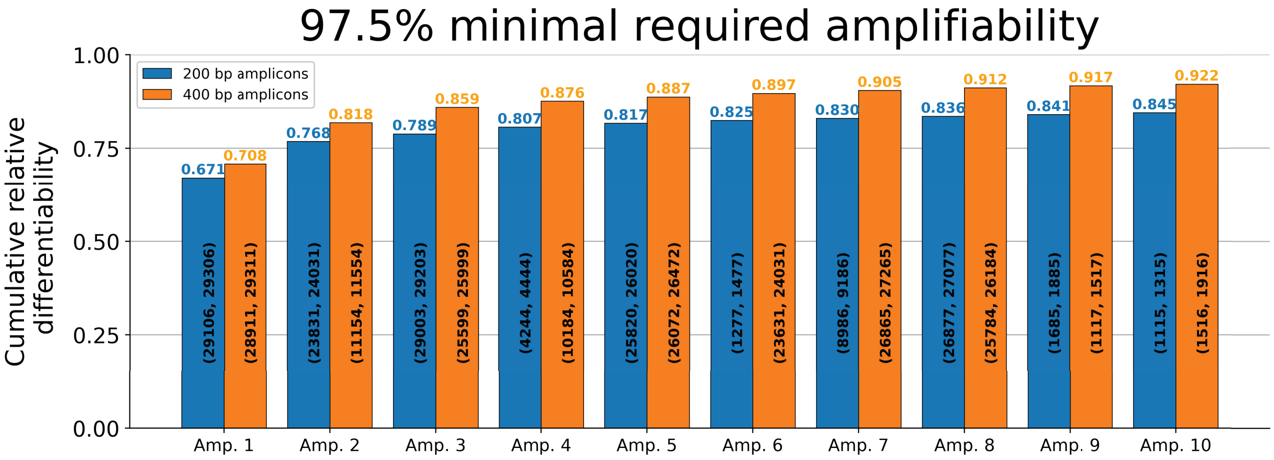

Figure S4: Cumulative relative differentiability of ten best amplicons (both 200 and 400 widths) found by AmpliDiff with the requirement of at least 97.5% amplifiability

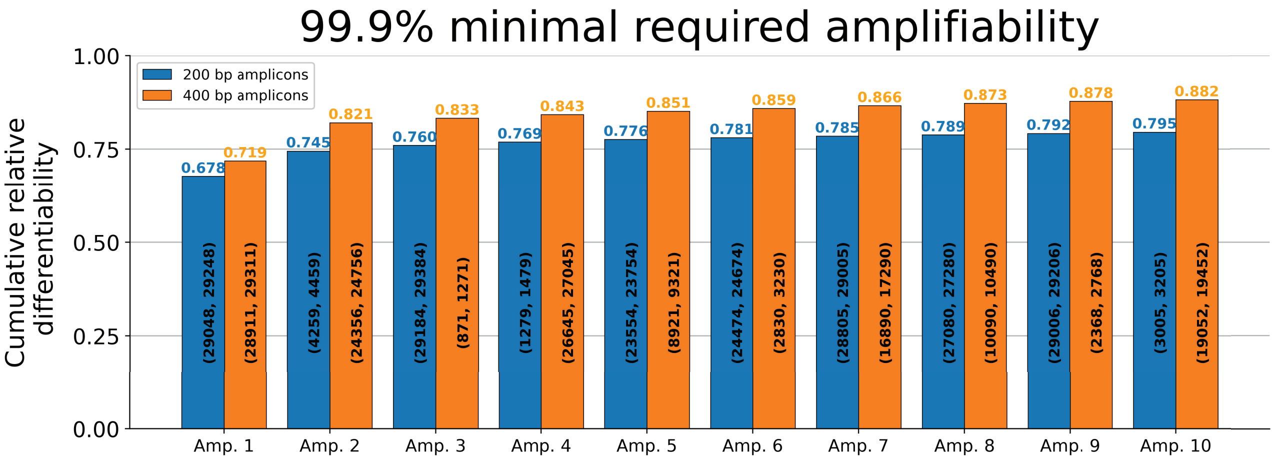

Figure S5: Cumulative relative differentiability of ten best amplicons (both 200 and 400 widths) found by AmpliDiff with the requirement of at least 99.9% amplifiability

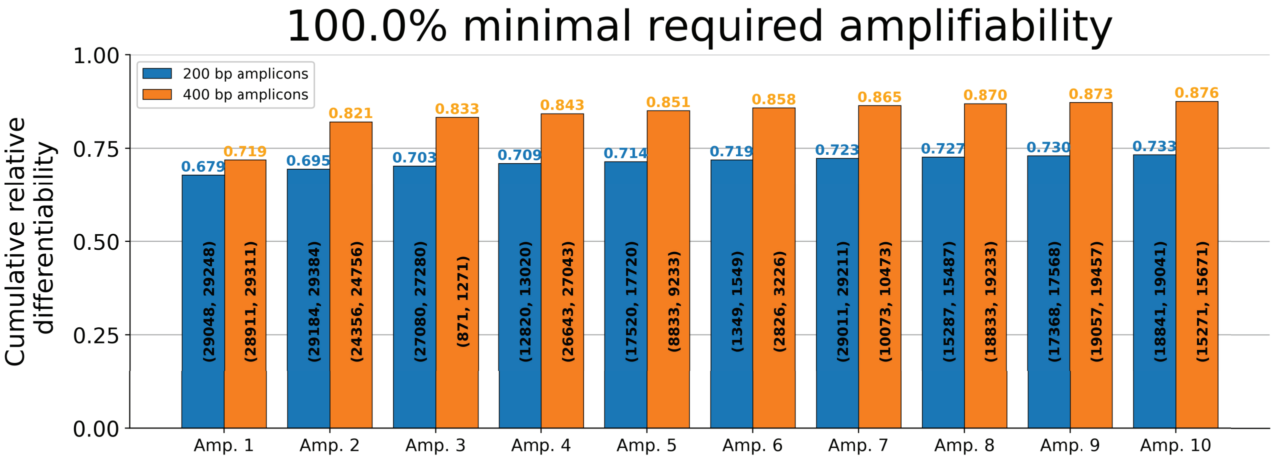

Figure S6: Cumulative relative differentiability of ten best amplicons (both 200 and 400 widths) found by AmpliDiff with the requirement of 100% amplifiability

**Amplicon width**	**200**						**400**					
**Minimal amplifiability**	90%	92.5%	95%	97.5%	99.9%	100%	90%	92.5%	95%	97.5%	99.9%	100%
Amplicon 1	1F, 1R	1F, 1R	1F, 1R	1F, 1R	7F, 6R	9F, 6R	1F, 1R	1F, 1R	1F, 1R	1F, 1R	3F, 4R	3F, 6R
Amplicon 2	1F, 1R	1F, 1R	1F, 1R	4F, 3R	11F, 5R	11F, 4R	1F, 1R	1F, 1R	1F, 1R	2F, 2R	5F, 3R	7F, 3R
Amplicon 3	2F, 2R	2F, 2R	2F, 2R	3F, 3R	9F, 4R	7F, 5R	1F, 1R	1F, 1R	1F, 1R	1F, 1R	5F, 5R	6F, 7R
Amplicon 4	1F, 1R	1F, 1R	1F, 1R	1F, 1R	9F, 9R	3F, 8R	2F, 2R	2F, 2R	2F, 2R	2F, 2R	1F, 5R	1F, 7R
Amplicon 5	1F, 1R	1F, 1R	1F, 1R	2F, 2R	4F, 6R	13F, 4R	1F, 1R	1F, 1R	1F, 1R	1F, 1R	5F, 5R	5F, 9R
Amplicon 6	1F, 1R	1F, 1R	1F, 1R	1F, 1R	4F, 2R	2F, 4R	1F, 1R	1F, 1R	1F, 1R	1F, 1R	5F, 6R	5F, 6R
Amplicon 7	1F, 1R	1F, 1R	1F, 1R	1F, 1R	2F, 9R	4F, 7R	2F, 2R	2F, 2R	2F, 2R	2F, 2R	4F, 2R	3F, 9R
Amplicon 8	2F, 2R	2F, 2R	2F, 2R	2F, 2R	5F, 5R	3F, 5R	1F, 1R	1F, 1R	1F, 1R	3F, 3R	4F, 4R	10F, 8R
Amplicon 9	1F, 1R	1F, 1R	1F, 1R	2F, 2R	3F, 5R	5F, 7R	1F, 1R	1F, 1R	1F, 1R	1F, 1R	3F, 7R	9F, 3R
Amplicon 10	1F, 1R	1F, 1R	1F, 1R	1F, 1R	12F, 7R	10F, 5R	1F, 1R	1F, 1R	1F, 1R	1F, 1R	7F, 2R	5F, 5R

Table S1: Number of primers corresponding to every amplicon found by AmpliDiff for different amplicon widths and minimal required amplifiabilities. F denotes the number of forward primers and R is the number of reverse primers.

The effect of sublineages on abundance estimations

To investigate the effect that sublineages of the Omicron lineage have on abundance estimations, we have looked at what happens when we aggregate lineages. To this end, for every non-recombinant direct sublineage of B.1.1.529 we have aggregated all of the sublineages (e.g. lineages starting with B.1.1.529.1 are all aggregated), and re-calculated the errors and MASEs for each of the experiments. The errors per lineage (including only lineages that occur in the reference set) are shown in Figure S7, where it can be seen that most errors are due to wrongly estimating the abundances of B.1.1.529 sublineages. In addition, Figure S8 shows the MASEs for the different experiments. In contrast to the results at the full lineage resolution, we now see that amplicon-based MASEs are much closer to the errors made by WGS, with 10 amplicons consistently outperforming WGS in both datasets under both amplicon widths. Hence, if the objective is to distinguish between direct sublineages of the B.1.1.529 lineage, the 10 amplicons found by AmpliDiff are able to do so.

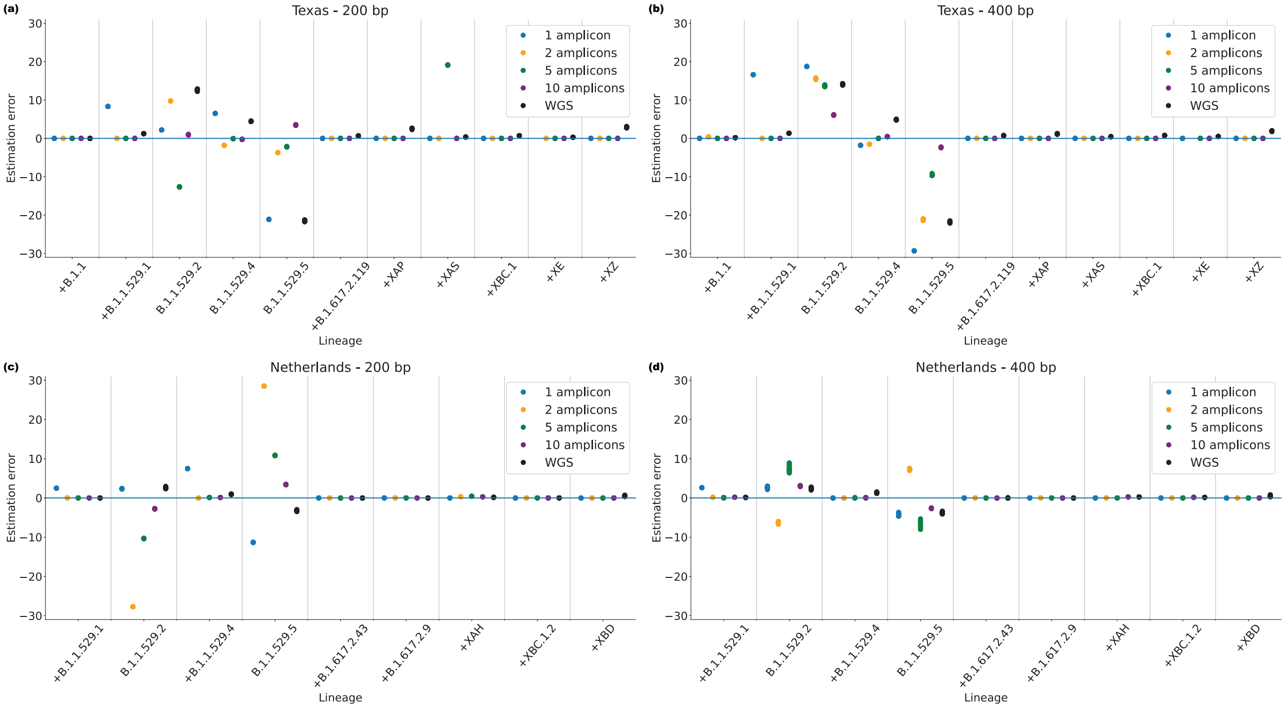

Figure S7: Estimation errors in for both the amplicon-based approach (1, 2, 5, 10 amplicons) and the whole genome-based approach using sequencing fragments of lengths 200 and 400 in both datasets. The x-axes show the lineages (cut-off at most 3 sublineage levels) where a lineage prefixed by a '+' character indicates a lineage that was not present in the simulation sample, and the y-axis is the estimation error.

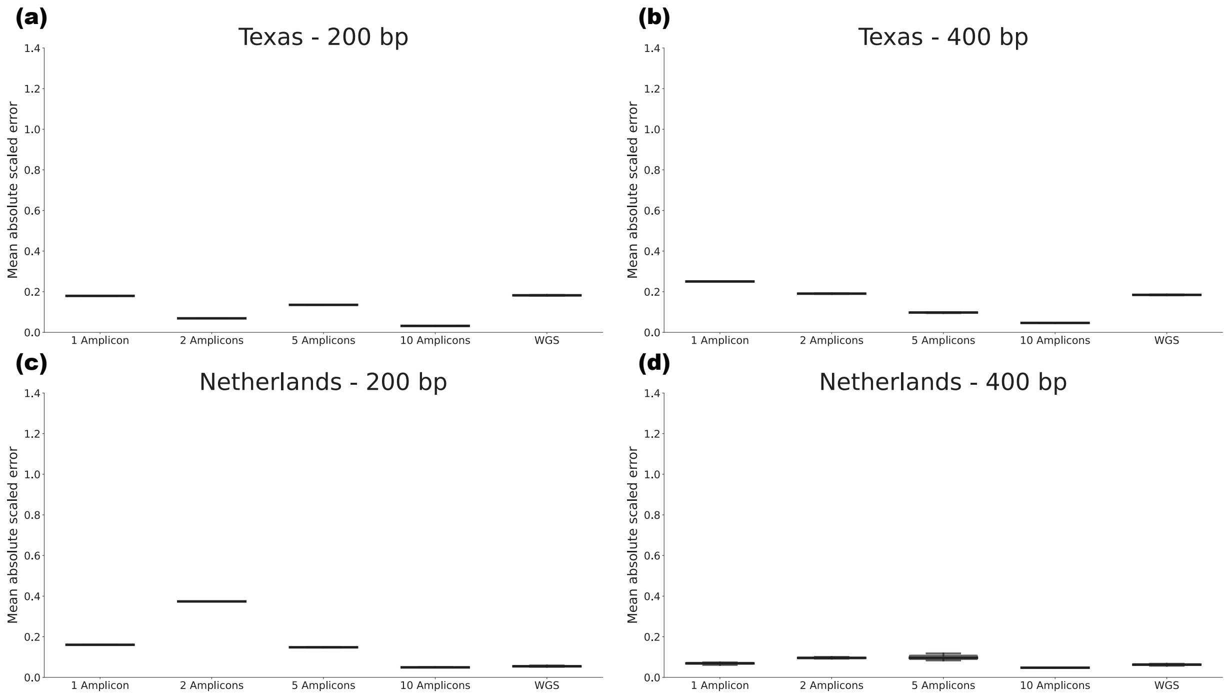

Figure S8: Mean absolute scaled errors of abundance estimations in both datasets for both amplicon widths using varying numbers of amplicons. **(a)** Texas dataset using amplicons of width 200. **(b)** Texas dataset using amplicons of width 400. **(c)** Netherlands dataset using amplicons of width 200. **(d)** Netherlands dataset using amplicons of width 400.

Primer binding results

(a)

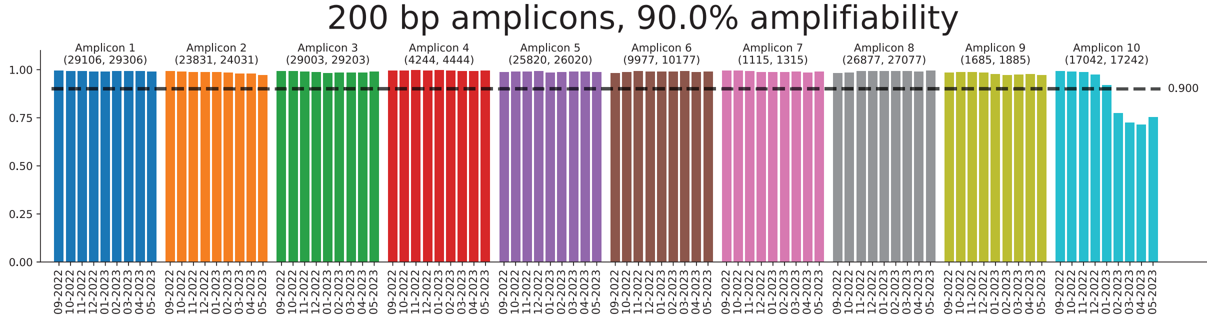

(b)

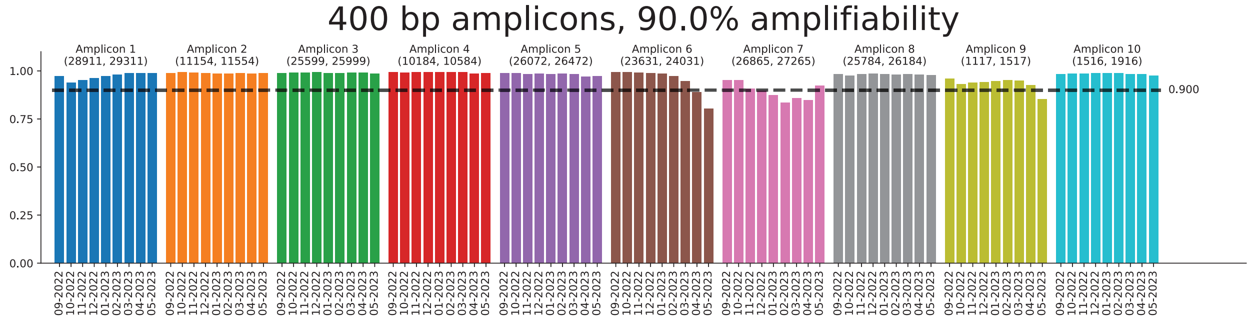

Figure S9: Fraction of genomes per month in which amplicons bind. **(a)** Amplifiability of 200 width amplicons generated with a 90% amplifiability requirement, in the "out of sample" genomes from September 2022 to May 2023. **(b)** Amplifiability of 400 width amplicons generated with a 90% amplifiability requirement, in the "out of sample" genomes from September 2022 to May 2023.

(a)

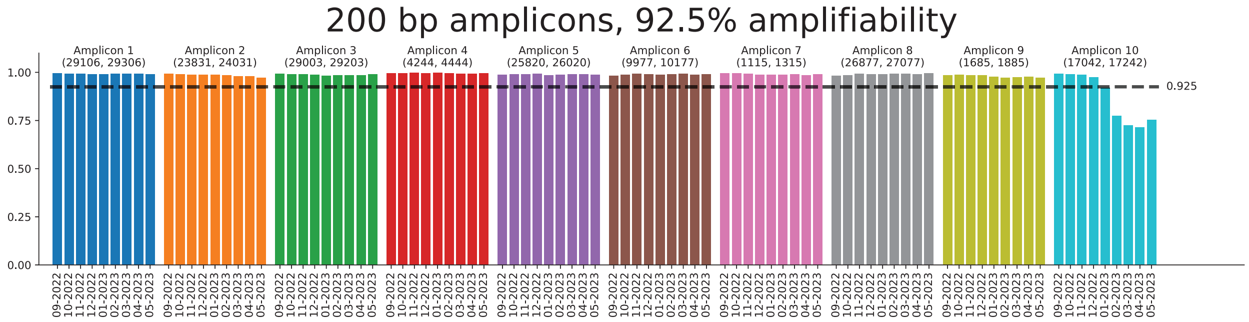

(b)

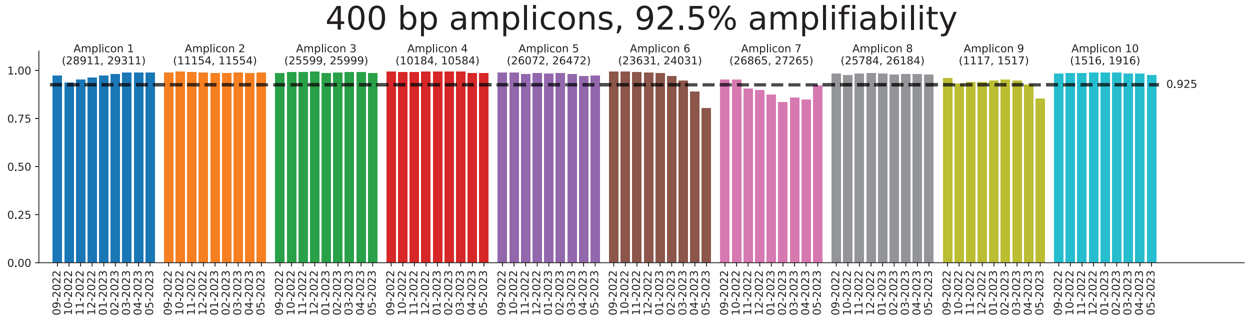

Figure S10: Fraction of genomes per month in which amplicons bind. **(a)** Amplifiability of 200 width amplicons generated with a 92.5% amplifiability requirement, in the "out of sample" genomes from September 2022 to May 2023. **(b)** Amplifiability of 400 width amplicons generated with a 92.5% amplifiability requirement, in the "out of sample" genomes from September 2022 to May 2023.

(a)

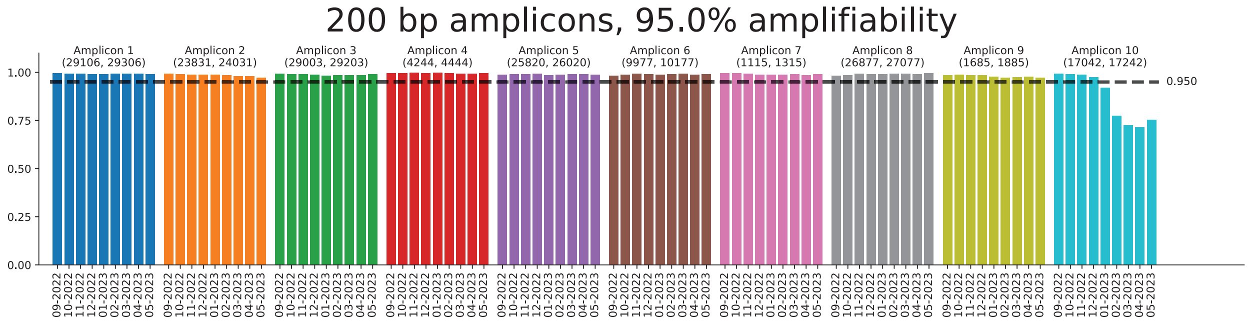

(b)

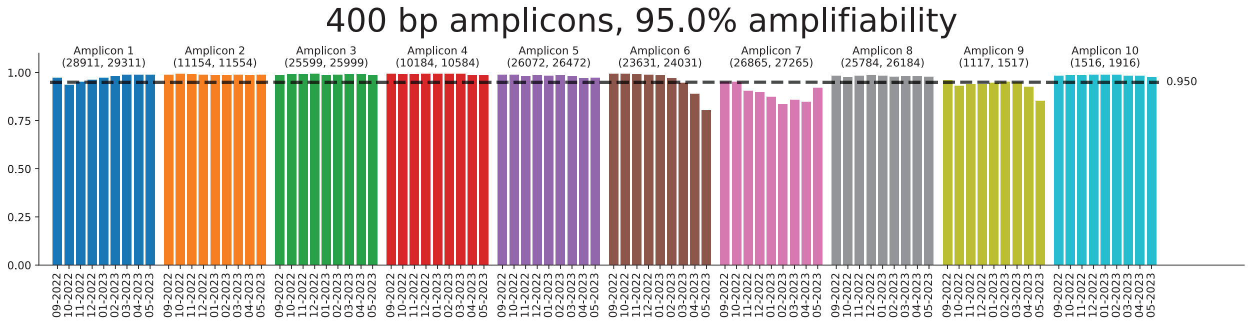

Figure S11: Fraction of genomes per month in which amplicons bind. **(a)** Amplifiability of 200 width amplicons generated with a 95% amplifiability requirement, in the "out of sample" genomes from September 2022 to May 2023. **(b)** Amplifiability of 400 width amplicons generated with a 95% amplifiability requirement, in the "out of sample" genomes from September 2022 to May 2023.

(a)

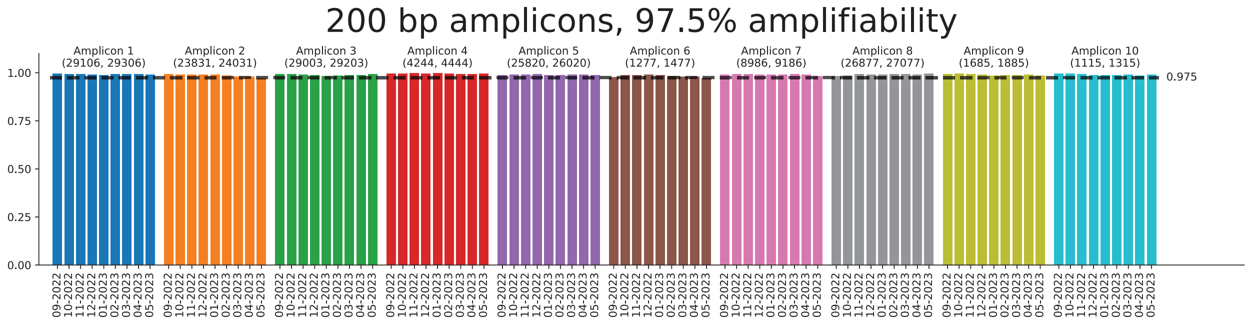

(b)

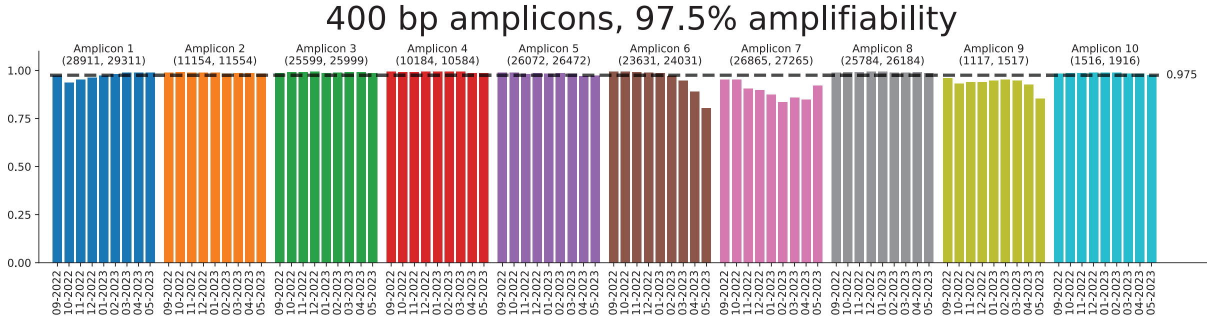

Figure S12: Fraction of genomes per month in which amplicons bind. **(a)** Amplifiability of 200 width amplicons generated with a 97.5% amplifiability requirement, in the "out of sample" genomes from September 2022 to May 2023. **(b)** Amplifiability of 400 width amplicons generated with a 97.5% amplifiability requirement, in the "out of sample" genomes from September 2022 to May 2023.

(a)

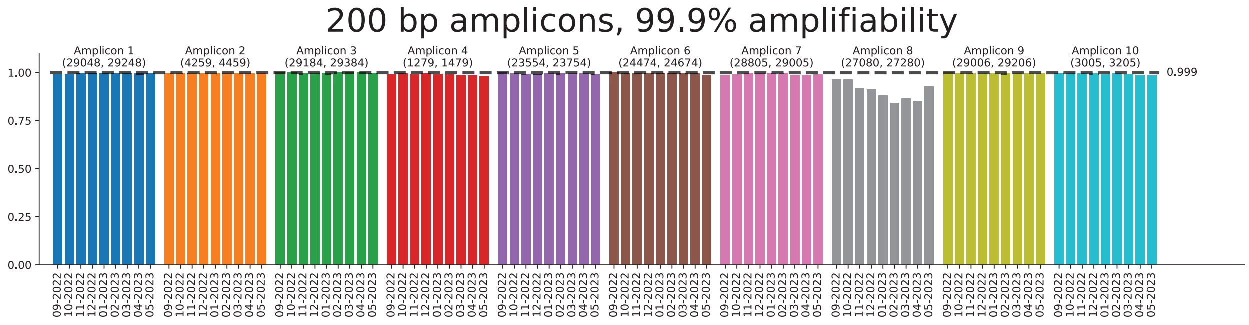

(b)

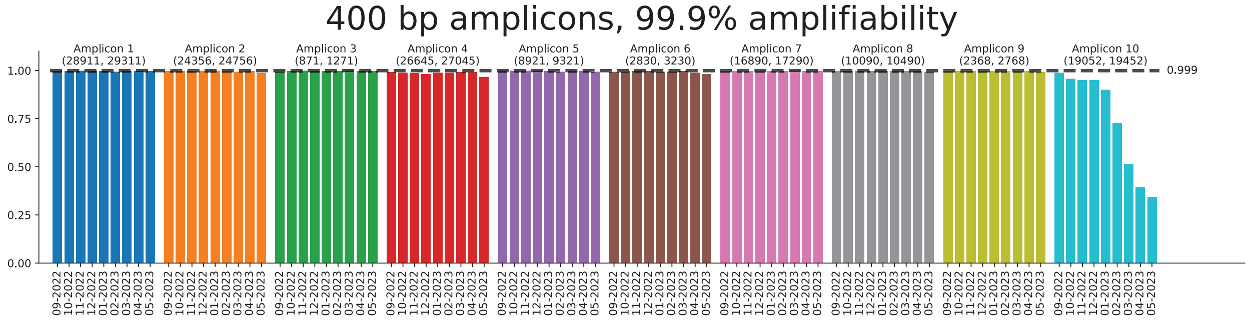

Figure S13: Fraction of genomes per month in which amplicons bind. **(a)** Amplifiability of 200 width amplicons generated with a 99.9% amplifiability requirement, in the "out of sample" genomes from September 2022 to May 2023. **(b)** Amplifiability of 400 width amplicons generated with a 99.9% amplifiability requirement, in the "out of sample" genomes from September 2022 to May 2023.

(a)

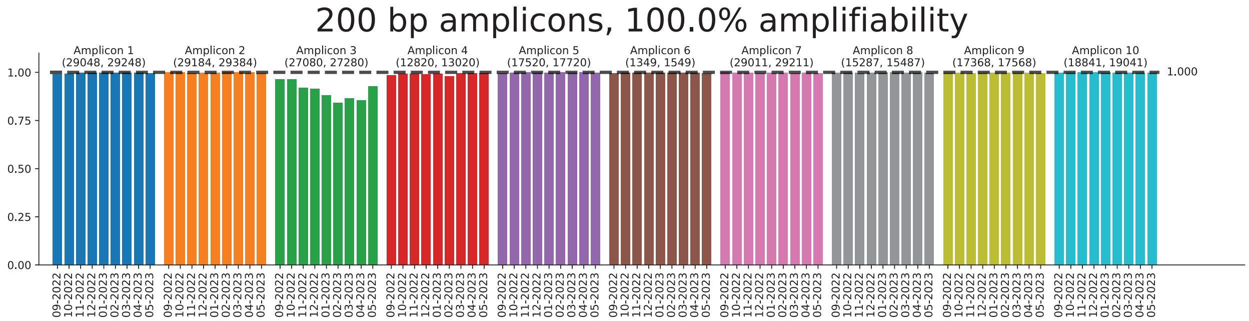

(b)

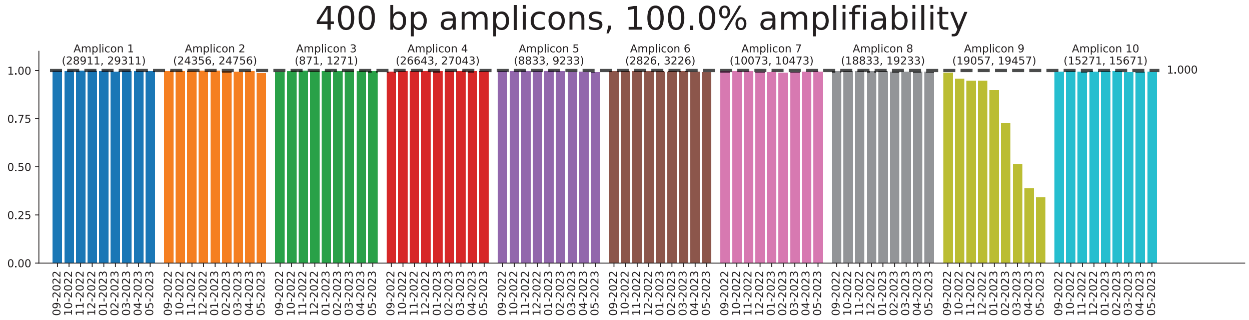

Figure S14: Fraction of genomes per month in which amplicons bind. **(a)** Amplifiability of 200 width amplicons generated with a 100% amplifiability requirement, in the "out of sample" genomes from September 2022 to May 2023. **(b)** Amplifiability of 400 width amplicons generated with a 100% amplifiability requirement, in the "out of sample" genomes from September 2022 to May 2023.

Computational requirements and complexity

Table S2 shows the average running time (over 15 runs) of AmpliDiff on 1500, 2000 and 2500 genomes with a minimal required amplifiability of 95%. The runtime of every step appears to increase with the number of included reference genomes.

To get insights in the theoretical computation time of AmpliDiff, we assume the determining the feasibility of a primer of length k only depends on the length of the primer such that it is equal to $O(f(k))$. Since the primer database construction needs to parse every k-mer (with k equal to the primer length) in each of the reference genomes, this yields a theoretical runtime of $O(|\mathcal{G}| \times |g^*| \cdot f(k))$ where $|\mathcal{G}|$ is the number of included reference genomes and $|g^*|$ is the length of the largest genome.

Determining the feasible amplicons is done by considering every window of the amplicon size (either 200 or 400 bp here), and calculating the number of misalignment characters. This can be achieved in $O(|\overline{g}|)$ time for every multiple aligned genome $\overline{g} \in \mathcal{G}$ as every character has to be considered exactly once. Calculating the cumulative number of misalignment characters can also be done in $O(|\overline{g}|)$ per genome which results in a theoretical runtime of $O(|\mathcal{G}| \cdot |\overline{g}|)$ for determining the feasibility of amplicons. In a similar fashion, calculating the amplicon differentiability is done by moving a window with a size equal to the amplicon length over all pairs of genomes of different class and calculating the number of positions where a pair disagree, which yields a runtime of $O(|\mathcal{G}|^2 \cdot |\overline{g}|)$.

Although it is difficult to give a theoretical runtime for the greedy amplicon picking algorithm, we can argue that it is likely NP-hard due to the primer feasibility and minimization problems, which are closely related to the set cover problem. Therefor, it is reasonable to assume that this part of the algorithm has an exponential runtime (in the variables of the problem formulation), which explains the growth in runtimes observed in Table S2.

	**200 bp width**				**400 bp width**			
**Task**	1500	2000	2500	2749*	1500	2000	2500	2749*
Constructing primer database	390s (119s)	682s (292s)	695s (179s)	673s	446s (131s)	548s (118s)	695s (178s)	818s
Determining feasible amplicons	100s (22s)	123s (23s)	164s (42s)	144s	96s (22s)	123s (29s)	163s (35s)	142s
Calculating amplicon differentiability	151s (36s)	274s (30s)	440s (102s)	526s	194s (33s)	351s (50s)	560s (127s)	602s
Greedy algorithm	10,825s (2,878s)	16,089s (3,062s)	23,239s (4,926s)	23,651s	8,941s (2,376s)	13,358s (3,085s)	22,121s (4,134s)	27,014s

Table S2: Average running time of AmpliDiff (95% minimal required amplifiability) on an HPC cluster using 200 GB RAM and 12 CPU cores for different numbers of included genomes. Standard deviations are shown in parentheses, with the exception of the full reference set running times for which we performed only a single run.

Primer selection criteria

- GC-content in range 40-60%
- Melting temperature in the range of 55-75degC
- Maximum of 2 A/T characters in final 3 nucleotides (3'-end)
- Maximum of 3 G/C characters in final 5 nucleotides (3'-end)
- Maximum run of 3 (i.e. 3 consecutive identical nucleotides)
- Maximum run of 2 duo nucleotides (e.g. ACAC)
- MFE threshold -5 (a proxy for the risk of hairpin formation)
- At most 10 self-complementary basepairs when compared to its own reverse complement (this is for the "worst" alignment)

Primer feasibility/optimization model

$$\max \sum_{(s,s')\in\mathcal{D}} y_{(s,s')} - \beta \cdot |\mathcal{D}| \cdot Q \tag{1}$$

$$\text{s.t.} \tag{2}$$

$$z_s \leq \sum_{p\in\mathcal{P}_s^f} x_p \qquad \forall s \in \mathcal{S} \tag{3}$$

$$z_s \leq \sum_{p\in\mathcal{P}_s^r} x_p \qquad \forall s \in \mathcal{S} \tag{4}$$

$$\sum_{s\in\mathcal{S}} z_s \geq \alpha|\mathcal{S}| \tag{5}$$

$$Q \geq \sum_{p\in\mathcal{P}^f} x_p \tag{6}$$

$$Q \geq \sum_{p\in\mathcal{P}^r} x_p \tag{7}$$

$$y_{(s,s')} \leq 0.5z_s + 0.5z_{s'} \qquad \forall (s,s') \in \mathcal{D} \tag{8}$$

$$T^+ \geq T_p x_p \qquad \forall p \in \mathcal{P}^f \cup \mathcal{P}^r \tag{9}$$

$$T^- \leq T_p(3 - 2x_p) \qquad \forall p \in \mathcal{P}^f \cup \mathcal{P}^r \tag{10}$$

$$T^+ - T^- \leq T^* \tag{11}$$

$$x_p + x_{p'} \leq C(p,p') \qquad \forall (p,p') \in (\mathcal{P}^f \cup \mathcal{P}^r) \times \big((\mathcal{P}^f \cup \mathcal{P}^r) \setminus p\big) \tag{12}$$

$$x_p \in \{0,1\} \forall p \in \mathcal{P}^f \cup \mathcal{P}^r \tag{13}$$

$$z_s \in \{0,1\} \forall s \in \mathcal{S} \tag{14}$$

$$T^+ \geq 0 \tag{15}$$

$$T^- \geq 0 \tag{16}$$

Model variables and parameters

Variables:

- x_p : binary variable equal to 1 if primer $p \in \mathcal{P}$ is selected
- z_s : binary variable equal to 1 if sequence s has at least one binding forward and one binding reverse primer

- $y_{(s,s')}$: binary variable equal to 1 if both sequences s and s' have at least one binding forward and one binding reverse primer
- T^* : continuous variable equal to the difference between the highest and lowest melting temperature of chosen primers
- Q : integer-valued variable equal to the number of primer pairs chosen

Variable sets:

- $\mathcal{D}$: set of all pairs of sequences of different class/lineage that can be differentiated by the current amplicon
- $\mathcal{S}$: set of all sequences
- $\mathcal{P}^f$: set of all forward primers
- $\mathcal{P}^r$: set of all reverse primers
- $\mathcal{P}^f_s$: set of forward primers corresponding to sequence s
- $\mathcal{P}^r_s$: set of all reverse primers corresponding to sequence s

Parameters:

- α : fraction of input genomes in which the amplicon must be amplifiable
- β : trade-off parameter between the number of included primer pairs and additional differentiability
- T^+ : maximum allowed melting temperature
- T^- : minimum allowed melting temperature
- T_p : approximate melting temperature of primer p
- $C(p, p')$: binary value equal to 1 if primers p and p' are predicted to form a primer-dimer pair

Constraints explanation

The first two sets of constraints set z_s equal to 1 if there primers are selected to amplify the amplicon in s (forward and reverse). The third constraint set enforces the amplicon amplifiability. Constraint sets 4 and 5 enforce that Q is equal to the number of primer pairs (i.e. the maximum of the number of forward and reverse primers). Since the objective minimizes Q it is only necessary to include these "greater or equal" constraints. Constraint set 6 enforces that $y_{(s,s')}$ is only set to 1 if both sequences s and s' satisfy amplifiability. Constraint sets 7 through

9 model the melting temperature constraints. Note that constraint set 8 uses $T^{-} \leq T_p(3-2x_p)$ which works as primer temperatures are generally in the range 40-70 and thus if x_p is not selected the constraint is redundant, and if x_p is selected then it sets T^{-} to at most T_p. Finally, constraint set 10 enforces that incompatible primers can not simultaneously be added to the solution.

This version of the model considers the optimization of the number of primer pairs needed for amplification of an amplicon, taking into account in which sequences the amplicon can be amplified. Note that in the feasibility check, the objective value is omitted as it is only required to check if a feasible solution exists. When the value of α is equal to 1, the model is simplified by removing the variables $y_{(s,s')}$ (and corresponding constraints) as these will be forced to equal 1. Additionally, the objective in this case is simply to minimize the number of required primer pairs Q.
